# Effect of Wild-Type *Shigella* Species and Attenuated *Shigella* Vaccine Candidates on Small Intestinal Barrier Function, Antigen Trafficking, and Cytokine Release

**DOI:** 10.1371/journal.pone.0085211

**Published:** 2014-01-09

**Authors:** Maria Fiorentino, Myron M. Levine, Marcelo B. Sztein, Alessio Fasano

**Affiliations:** 1 Mucosal Immunology and Biology Research Center, Massachusetts General Hospital, Charlestown, Massachusetts, United States of America; 2 Center for Vaccine Development and the Departments of Pediatrics and Medicine, University of Maryland School of Medicine, Baltimore, Maryland, United States of America; University of Chicago, United States of America

## Abstract

Bacterial dysentery due to *Shigella* species is a major cause of morbidity and mortality worldwide. The pathogenesis of *Shigella* is based on the bacteria's ability to invade and replicate within the colonic epithelium, resulting in severe intestinal inflammatory response and epithelial destruction. Although the mechanisms of pathogenesis of *Shigella* in the colon have been extensively studied, little is known on the effect of wild-type *Shigella* on the small intestine and the role of the host response in the development of the disease. Moreover, to the best of our knowledge no studies have described the effects of apically administered *Shigella flexneri* 2a and *S*. *dysenteriae* 1 vaccine strains on human small intestinal enterocytes. The aim of this study was to assess the coordinated functional and immunological human epithelial responses evoked by strains of *Shigella* and candidate vaccines on small intestinal enterocytes. To model the interactions of *Shigella* with the intestinal mucosa, we apically exposed monolayers of human intestinal Caco2 cells to increasing bacterial inocula. We monitored changes in paracellular permeability, examined the organization of tight-junctions and the pro-inflammatory response of epithelial cells. *Shigella* infection of Caco2 monolayers caused severe mucosal damage, apparent as a drastic increase in paracellular permeability and disruption of tight junctions at the cell-cell boundary. Secretion of pro-inflammatory IL-8 was independent of epithelial barrier dysfunction. *Shigella* vaccine strains elicited a pro-inflammatory response without affecting the intestinal barrier integrity. Our data show that wild-type *Shigella* infection causes a severe alteration of the barrier function of a small intestinal cell monolayer (a proxy for mucosa) and might contribute (along with enterotoxins) to the induction of watery diarrhea. Diarrhea may be a mechanism by which the host attempts to eliminate harmful bacteria and transport them from the small to the large intestine where they invade colonocytes inducing a strong inflammatory response.

## Introduction

Shigellosis is a leading cause of diarrheal disease, particularly in developing countries, estimated to have caused up to 1 million deaths per year between 1966–1997 [1]. Travelers and military personnel are at high risk of infection in the endemic countries. In the United States, there are approximately 14,000 laboratory-confirmed cases of shigellosis and an estimated 448,240 total cases that occur each year, according to the Center for Disease Control [2]. The peak incidence of shigellosis is among children 1 to 4 years of age, although no age groups are spared. Shigellosis can induce serious growth retardation [3]. Development of an efficacious vaccine against *Shigella* is a high priority for the protection of populations in developing countries, where the burden of *Shigella* causes much morbidity and claims many young lives. The same vaccines would also represent a potential tool to protect industrialized country travelers against the risk of travel shigellosis.

*S. flexneri* serotypes along with *S. sonnei* are responsible for most endemic disease in developing and transitional countries. *S*. *dysenteriae* type 1 is unique among *Shigella* in that it elaborates Shiga toxin (which exhibits potent neurotoxic, cytotoxic and enterotoxic activities), causes unusually severe clinical disease (including, rarely, hemolytic uremic syndrome) and is capable of large explosive outbreaks and true pandemic spread [4]. Two other enterotoxins are produced by *Shigella* strains and considerable evidence indicates that they are associated with the secretory diarrhea observed early in the clinical picture of shigellosis before the onset of dysentery (diarrheal stools with gross blood). These include *Shigella* enterotoxin 1 (ShET 1), restricted almost exclusively to the *S. flexneri* 2a serotype [5]–[8] and *Shigella* enterotoxin 2 (ShET2) which is found in all *Shigella* serotypes as it is encoded by genes located on the invasiveness plasmid [9], [10].

Because the ingestion of as few as 10 organisms is sufficient to cause clinical shigellosis [11], this is a highly contagious infectious disease that is readily transmitted by fecal-oral contact.

There's a wealth of literature available describing the mechanisms by which *Shigella* cause dysentery. Most of these studies have focused on *S. flexneri* and *S. dysenteriae* 1 in animal models, including macaques, to assess the host response to infection with wild-type strains and immunogenicity and protection elicited by orally administered live attenuated vaccine candidates [12]–[20]. A few studies have also been performed in humans immunized and/or challenged with wild-type *Shigella* [21]–[26], using a model of human colonic explants [27], [28] or mainly focusing on undifferentiated crypt-like cells [29], [30]. While the mechanisms of pathogenesis of *Shigella* in the colon have been extensively studied, very limited and scattered information is available on the effect of wild-type *Shigella* on the small intestine and the role of the host response to infection of the small intestine itself in the development of the disease [28], [31], [32]. Similarly, most of the studies available on the effects of *Shigella* auxotrophic mutants on epithelial cells have been performed on basolaterally infected Caco2 cells, T84 or Hela cells and/or animal model colonic explants [30], [33]–[35].

The aim of this study was therefore to assess the coordinated human epithelial responses, at both the functional and immunological levels evoked by apically administered strains of wild-type *Shigella* and candidate vaccines, by studying changes in Caco2 cell monolayer barrier integrity, tight junction function and secretion of pro-inflammatory cytokines.

## Materials and Methods

### Cell culture

Human Caco-2 intestinal epithelial cells [HTB-37, American Type Culture Collection (ATCC), Rockville, MD] were routinely grown in Dulbecco's Minimum Essential Medium (DMEM) supplemented with 20% fetal bovine serum. Monolayers (passages 22-35) were grown on 1.12 cm^2^ permeable polyester filters with 0.4 um pore size (Corning, Lowell, MA) and utilized after 14–21 days until having reached a confluent, polarized and differentiated state.

### Bacteria strains and growth conditions

*S. flexneri* 2a strain 2457T and *S. dysenteriae* 1 1617 are wild-type strains [36], [37]. Bacteria were routinely grown at 37°C in Luria Bertani (LB) overnight. Two hundred microliters of a stationary phase culture were used to inoculate 5 ml of DMEM, and the bacteria were grown in a shaking incubator for approximately 2 h at 37°C to mid-exponential phase (OD_600nm_ = 0.3). CVD 1208S and CVD 1256 are live, attenuated, oral vaccine candidates *de novo* constructed from their parental strains *Shigella flexneri* 2a strain 2457T and *Shigella dysenteriae* 1 1617, respectively. These attenuated strains harbor deletions in the guanine nucleotide synthesis pathway and in the genes encoding *Shigella* enterotoxins [30], [38]. Vaccine strains were routinely grown on LB agar plates and in DMEM media supplemented with guanine [30]. The CVD 1208S strain used in this study has been reconstructed by using animal-free media [39].

### Generation of conditioned media and heat-killed cultures

Aliquots of overnight precultured bacteria were grown in DMEM for 3 hours to a final OD_600_ of 0.3. Cells were pelleted, and supernatants filter sterilized by passing through a 0.22-um pore size filter. Supernatants, hereafter referred to as ‘conditioned media’, were immediately used. Bacteria grown in DMEM for 3 hours as described above were heat-killed by boiling at 100°C for 30 minutes.

In both cases, the effectiveness of filtration and killing by heat was confirmed by lack of bacterial growth from 100 µl of this media plated onto agar plates and then incubated overnight at 37°C.

### Measurement of TEER

Transepithelial electric cell resistance (TEER) was used to monitor the integrity of the epithelial monolayer using a Millicel ERS Volt-ohm meter (World Precision Instruments, New Haven, CT). Monolayers with TEER values within ∼800–1200 Ω.cm^2^ were considered to have an appropriate barrier function and were used in the study.

Caco2 cells were infected apically with the bacteria suspension, heat-killed bacteria or bacterial supernatants at an inoculation ratio (MOI) of 500, 50 and 5 bacteria/epithelial cell (corresponding to 10^7^, 10^6^ and 10^5^ CFU) and incubated at 37°C. After 6 hours of incubation, cells were washed with PBS to remove non-adherent bacteria and treated with gentamicin. Monolayers were then incubated at 37°C overnight. TEER was measured at the time points indicated. Gentamicin-resistant intracellular bacteria counts were enumerated from representative infected monolayers after cell lysis for 30 min at 37°C with 1 ml 0.1% Triton X-100 in H_2_O, by serial 10-fold dilution in PBS and plating on LB or LB agar (data not shown).

### Cell viability

Caco2 cell monolayer viability after bacterial infection was assessed using a lactate dehydrogenase (LDH) secretion assay. The LDH secretion was measured from the cellular supernatant by a commercially available LDH assay kit (Cytotox 96, Promega, WI) and carried as described by the manufacturer's instructions. Lysis of the cells with 1% Triton X-100 served as positive control.

### Assessment of Caco2 Cell Monolayer Permeability

The permeability of the Caco2 cell monolayer was evaluated by measuring the flux of Fluorescein isothiocyanate (FITC)-Bovine Serum Albumin (BSA) with a molecular weight of 66 kDa (Sigma).

FITC-BSA was dissolved in P buffer (10 mM HEPES, pH 7.4, 1 mM sodium pyruvate, 10 mM glucose, 3 mM CaCl_2_, 145 mM NaCl) or P/EGTA buffer [(10 mM HEPES, pH 7.4, 1 mM sodium pyruvate, 10 mM glucose, 145 mM NaCl, 2 mM ethylene glycol-bis(ß-aminoethyl ether)-*N,N,N*',*N*' -tetraacetil acid (EGTA)].

Briefly, the apical surface of Caco2 cell monolayers was infected with either wild-type *S. flexneri* 2a or *S*. *dysenteriae* 1, CVD 1208S or CVD 1256 for 6 h, washed, treated with gentamicin and incubated at 37°C overnight. In order to measure the paracellular flux, the apical and basolateral cell culture media were replaced with P buffer containing FITC-BSA (10 mg/ml) and P buffer alone, respectively. P/EGTA buffer containing FITC-BSA (10 mg/ml) and P/EGTA buffer were used as positive controls. After incubation for 4 h, the amounts of FITC-BSA in the basolateral media were measured with a fluorometer (excitation at 492 nm and emission at 520 nm).

### Western blot (Triton X-100 soluble and insoluble fractions)

Bacteria were added to the apical surface of Caco2 cell monolayers at a MOI of 50∶1 for 4 hours at 37°C. Then cells were washed with PBS and incubated with DMEM supplemented with gentamicin overnight at 37°C. Cells were harvested at 6 and 24 hours post-infection and Triton X-100-soluble and -insoluble protein fractions were prepared. Monolayers were harvested on ice in lysis buffer [1% Triton X-100, 100 mM NaCl, 10 mM HEPES, 2 mM EDTA, 4 mM Na3VO4, 40 mM NaF 200 mM PMSF, and a protease inhibitor cocktail (Complete Mini, Roche Molecular Biochemicals, Mannheim, Germany) and phosphatase inhibitors (Sigma, St. Louis, MO)]. Lysates were rotated at 4°C, 30 min, centrifuged (14000 g for 30 min at 4°C) and the supernatant suspension, representing the Triton X-soluble fraction, was collected. The remaining pellet was re-suspended in lysis buffer supplemented with 1% SDS and sonicated (5 W, 5 s) two-three times on ice. The resulting suspension was centrifuged (14000 g for 5 min at 4°C) and the supernatant, representing the Triton X-insoluble fraction, was collected. Samples were used immediately or stored at −80°C. Protein concentration was quantified by the Bradford method (Bio-Rad, Hercules, CA). Samples were electrophoresed through a 10–20% gradient SDS polyacrylamide gel and transferred onto polyvinylidene difluoride membranes (Millipore, Bedford, MA). Membranes were blocked in blocking buffer (Tris-buffered saline, 0.1% Tween 20, 5% BSA), for 1 hour at room temperature. The blots were incubated overnight at 4°C with mouse anti-occludin diluted in blocking buffer. After washing, membranes were incubated for 1 hour at room temperature with the appropriate secondary antibody diluted in blocking buffer. The hybridized band was detected by chemiluminescence using an ECL kit (Amersham) according to the manufacturer's instructions. Membranes were stripped and reprobed (Blot restore solution, Millipore) for the detection of phosphothreonine followed by actin that served as the loading control. Band intensity was normalized to actin and quantitated by densitometry using Image J software (National Institutes of Health). Data represent the average of two separate experiments.

### Cytokine assay

Media from both upper and lower transwell compartments were collected at 24 h postinfection. Samples were centrifuged at 2,000 rpm for 10 minutes to remove any residual cells or debris. Supernatants were stored at −80° until evaluated for cytokine secretion by Meso Scale Discovery (Rockville) technology, using a Human pro-inflammatory 7Plex Ultrasensitive Plate. Kits were run according to the manufacturer's instructions, with the exception of sample collection and processing as described above. Samples were run in duplicate.

### Immunostaining

Cell monolayers on chamber slides were washed three times with PBS and fixed in PFA 4%/PBS or methanol for 20 min at room temperature or −20°C, respectively.

Cells were then blocked with 2% PBS diluted normal goat serum/1% BSA (blocking solution) for 30 min and incubated with blocking solution-diluted primary antibodies overnight at 4°C (anti-ZO-1, 1∶100; anti-claudin 1, 1∶1000).

After 3 washes with PBS, monolayers were incubated with 488 nm or 555 nm Alexa Fluor-conjugated secondary antibodies (1∶800 in blocking solution) at room temperature for 1 h in the dark. Monolayers were washed with PBS and nuclei stained with DAPI (1∶1000 in PBS) solution for 2 min at room temperature. Monolayers were analyzed with a Nikon Eclipse TE2000-E fluorescent microscope.

### Antibodies

Rabbit anti-claudin-1(JAY-8, cat # 51-9000), mouse anti-occludin (OC-3F10, cat # 33-1500), mouse anti-ZO-1 (1A12, cat # 339100) and rabbit anti-phosphothreonine (ZPT1cat # 718200) antibodies were purchased from Invitrogen (Camarillo, CA). Mouse anti-actin protein antibody (5C, cat # 82353) was purchased by Thermo Fisher Scientific (IL).

### Statistics

Data were analyzed by using GraphPad (San Diego, CA) software. Comparisons among time points within the same treatment group were made by two-way ANOVA. Comparisons among groups were made by one-way ANOVA. Differences were considered statistically significant if “*p*” values were <0.05.

## Results

### *Shigella* spp. adversely affect epithelial monolayer Trans-Epithelial Electric Resistance (TEER)

*Shigella* spp. are highly invasive bacteria affecting the mucosal layer of the intestine. To evaluate the effects of the interaction between the pathogens and the host on the mucosal barrier integrity and function, we apically infected Caco2 monolayers with bacteria at different inocula and monitored Transepithelial Electric Resistance (TEER) as a measure of tight junction function.

**Figure 1:**
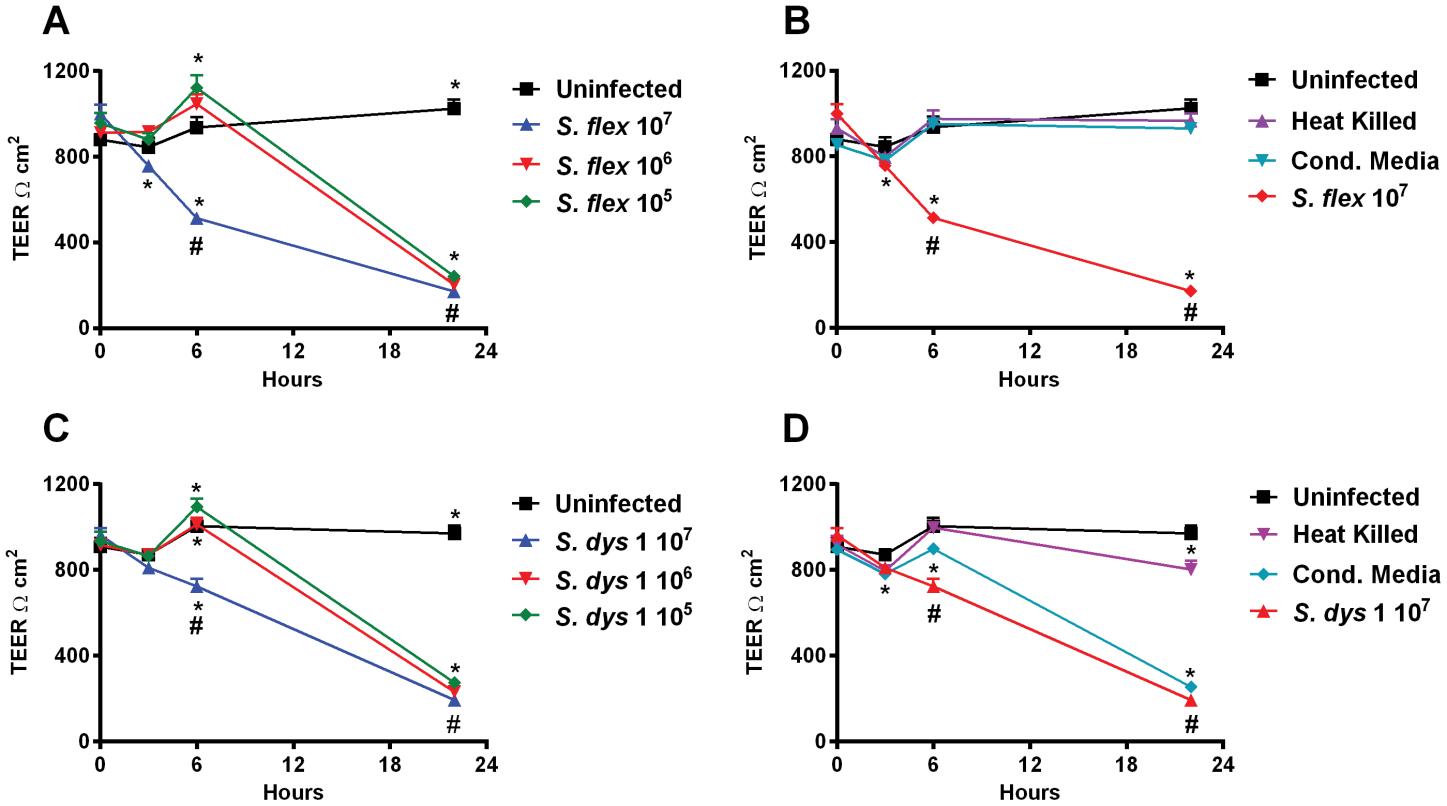
Wild type *Shigella* induce TEER decrease in Caco2 cell monolayers independent of MOIs. **A**. TEER responses to wild-type *S. flexneri* 2a applied at three different inocula. **B**. TEER responses to *S. flexneri* 2a heat-killed bacteria and culture supernatants (Cond. Media). **C**. TEER responses to wild-type *S. dysenteriae* 1 applied at three different inocula. **D**. TEER responses to *S. dysenteriae* 1 heat-killed bacteria and culture supernatants. Data are expressed as means ± SEM for triplicate samples for all conditions tested. * and # indicate statistically significant differences vs. t = 0 and vs. the uninfected control at the same time point, respectively; *p*<0.05 (ANOVA). These results are representative of 3 experiments with similar results.

Caco2 cell monolayers were challenged with increasing amounts of wild-type *Shigella flexneri* 2a or *S. dysenteriae* 1. As shown in Fig. 1 (A, C) we found that wild-type *Shigella* species induced a marked drop in TEER in the epithelial cell monolayer at all inocula evaluated (10^5^, 10^6^ and 10^7^ CFU/well, corresponding to MOIs of 5, 50 and 500∶1 bacteria:epithelial cell, respectively). At 22 hours post infection with *S. flexneri* 2a we recorded the almost total disruption of the monolayer electric resistance, being TEER values comparable to those of initial cell seeding when no tight-junctions are still formed between cells (242±22.6 Ω.cm^2^, 203±3.9 Ω.cm^2^ and 171±2.6 Ω.cm^2^ for 10^5^, 10^6^ and 10^7^ CFU/monolayer, respectively). *S. dysenteriae*-1 TEER values at 22 hour were similarly low (274±15.4 Ω.cm^2^, 231±4.6 Ω.cm^2^ and 193±6.7 Ω.cm^2^) suggesting that both wild-type *Shigella* species greatly affected monolayer barrier function integrity independent of the bacteria load.

Interestingly, and unlike our observations with *Salmonella* Typhi [40], we detected an increase in TEER in the initial few hours of infection (6 hours), when the monolayers were treated with bacteria at 10^5^ and 10^6^ CFU/monolayer. The greatest increase for both *Shigella* strains was observed when a 10^5^ bacterial load was applied, for which we recorded TEER values of 1121±59.9 Ω.cm^2^ for *S*. *flexneri* 2a and 1094±38.0 Ω.cm^2^ for *S*. *dysenteriae* 1, compared to the uninfected monolayer (936±49.1 Ω.cm^2^ and 1004±38.7 Ω.cm^2^, respectively). Conversely, at the highest bacteria load, TEER decreased at 6 hours of about 50% and 25% of the initial value for *S. flexneri* 2a and *S*. *dysenteriae* 1, respectively (Fig. 1A, C).

At the end of the experiments, aliquots of lysates from the monolayers were plated on LB-agar to monitor for intracellular bacteria. Over 250 CFUs/monolayer were isolated following infection with *S. flexneri* 2a at 10^5^, showing the ability of this pathogen to efficiently invade cells at their apical pole. Surprisingly, we observed much less bacteria/monolayer after infection with *S. dysenteriae* 1 (≤50). As a further control, we plated on agar an aliquot of media from the upper compartment supernatant, containing bacteria and did not observe any difference between the two strains at 6 hours, confirming that the monolayer had been infected with a comparable bacterial load. Both attenuated strains CVD 1208S and CVD 1256 showed a very small number of bacteria/monolayer (<5).

Further, we tested wild-type heat-killed bacteria and bacteria culture supernatant to determine whether viable organisms are required to affect the integrity of the epithelial barrier. As shown in Fig. 1 B and D, both heat-killed *S. flexneri* 2a and *S. dysenteriae* 1, applied at the same titer as viable wild-type bacteria, failed to significantly decrease the TEER of Caco2 cell monolayers. Unlike *S. flexneri* 2a, conditioned media from *S. dysenteriae* 1 had a decreasing effect on TEER at 22 hours, comparable to that of live cultures (Fig. 1D).

### CVD 1208S and CVD 1256 strains designed for vaccine development, have an attenuated effect on epithelial barrier function

**Figure 2:**
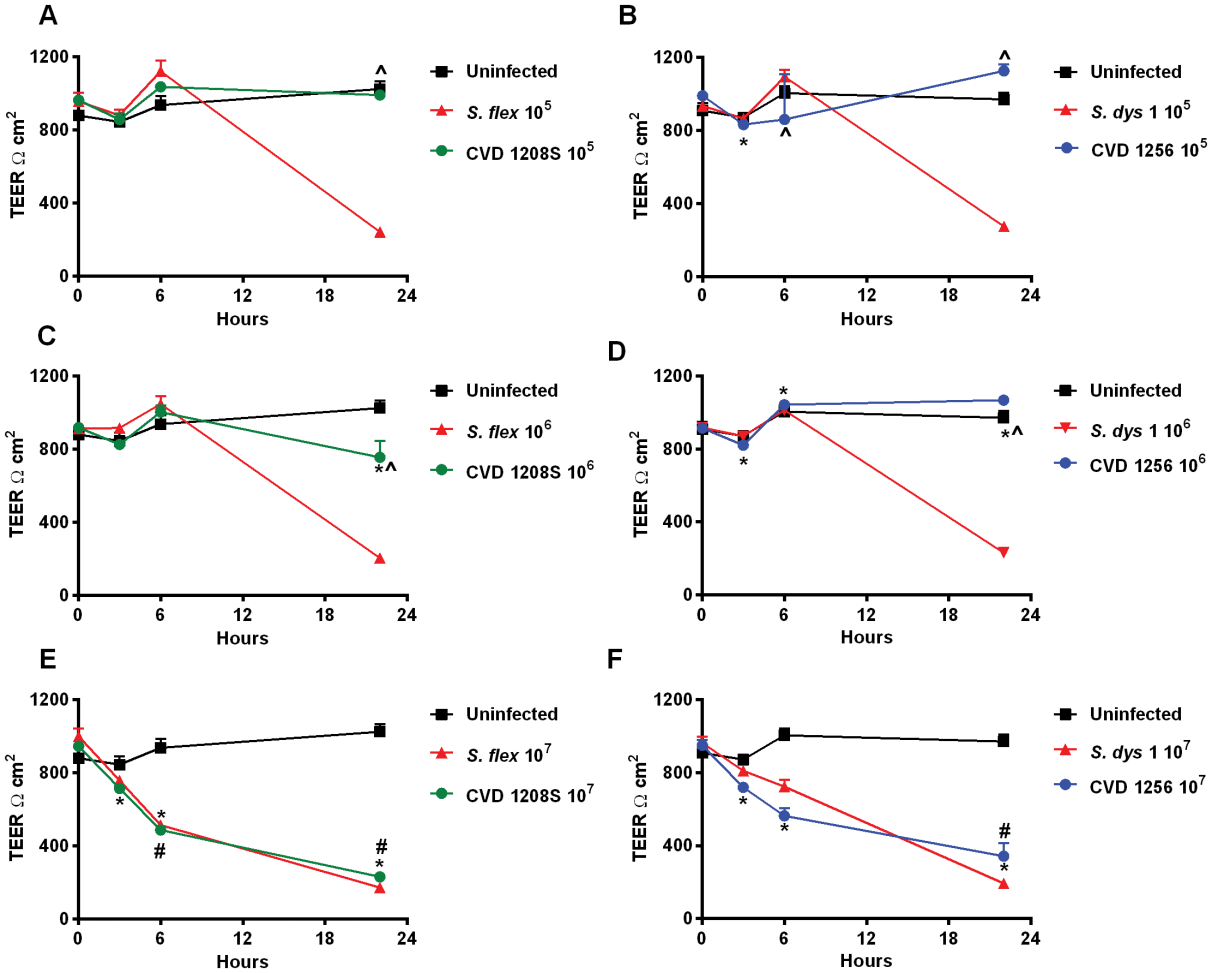
*Shigella* vaccine strains have attenuated effects on Caco2 monolayer barrier function, except at the highest inoculum. **A, C, E.** TEER of Caco2 cells infected with wild-type *S*. *flexneri* 2a and vaccine candidate *S. flexneri* 2a CVD 1208S applied apically at inocula of 10^5^, 10^6^ and 10^7^ CFU. **B, D, F.** TEER of Caco2 cells infected with wild-type *S*. *dysenteriae* 1 and attenuated strain *S. dysenteriae* 1 CVD 1256 applied apically at inocula of 10^5^, 10^6^ and 10^7^ CFU. Data are expressed as means ± SEM for triplicate samples for all conditions tested. * and ∧ indicate statistically significant differences vs. t = 0 and vs. wild-type *Shigella* at the same inoculum and time point, respectively. # denotes statistically significant differences vs. the uninfected control at the same time point; *p*<0.05 (ANOVA). Statistical analysis results are shown only for the attenuated strains. Please, refer to Figure 1 for wild-type strains. These results are representative of 3 experiments with similar results.

To evaluate their potential use as candidate vaccines, the effect of *S. flexneri* 2a and *S. dysenteriae* 1 attenuated strains was assessed on Caco2 monolayers. Caco2 cells were infected with CVD 1208S (Fig. 2A, C, E) or CVD 1256 (Fig. 2B, D, F) attenuated vaccine strains, in parallel with wild-type *S. flexneri* 2a and *S. dysenteriae* 1 strains, respectively. Vaccine strains were applied to Caco2 monolayers at the same titer as the wild-type bacteria. Both CVD 1208S and CVD 1256 attenuated strains induced a significant decline in TEER only when applied at the highest load (Fig. 2E, F). However, as the bacterial load was diminished, the milder effect on barrier function of the vaccine candidates compared to the wild-type became more apparent (Fig. 2C, D) with nearly no effect when applied at 10^5^ (Fig. 2A, B).

### Wild-type *Shigella* but not vaccine strains infection has a mild effect on Caco2 monolayer viability

**Figure 3:**
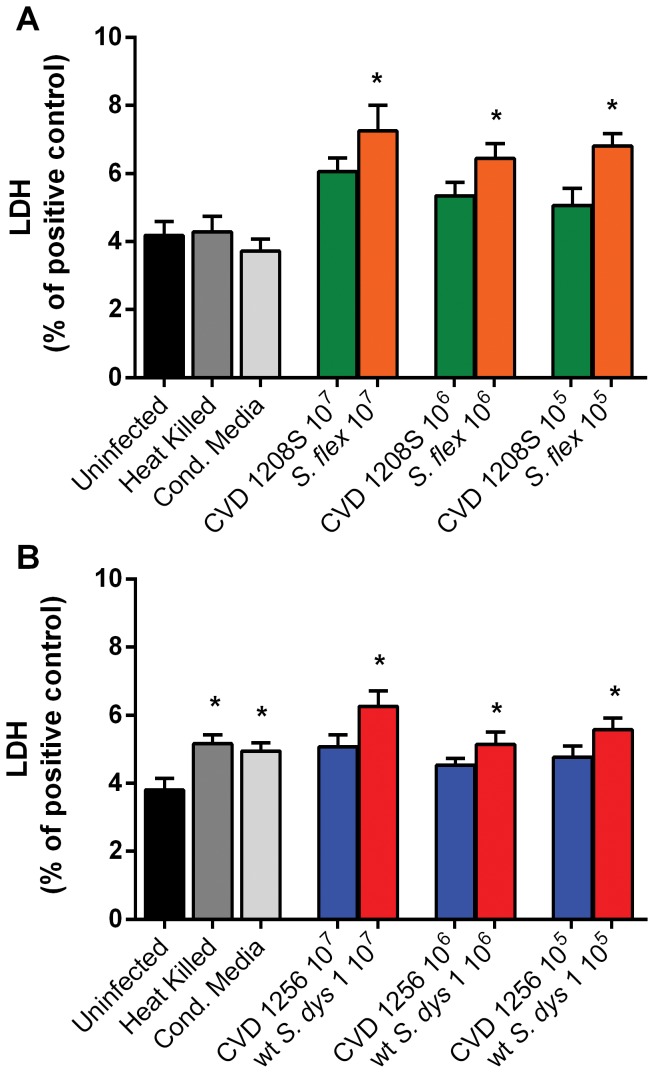
Wild type *Shigella*, but not vaccine strains, moderately affect Caco2 monolayer viability. **A**. LDH release after infection with wild-type *S. flexneri* 2a or its vaccine strain CVD 1208S at different inocula. **B**. LDH release after infection with wild-type *S. dysenteriae* 1 or its vaccine strain CVD 1256 applied at different inocula. Caco2 cells were infected with bacteria for 6 hours, washed and incubated overnight at 37°C. Apical supernatants were collected at 24 hours post-infection for LDH release measurement. Values represent LDH release from cells into the medium as a percentage of total LDH (Control). Data are expressed as means ± SEM for triplicate samples for all conditions tested. These results are representative of 3 experiments with similar results. *p<0.05 compared to uninfected cells (ANOVA).

To evaluate whether the disruption of the epithelial barrier function induced by *S. flexneri* 2a and *S. dysenteriae* 1 is mediated by enhanced apoptosis, the viability of the monolayer was assessed by measuring the levels of lactate dehydrogenase (LDH), released in the medium by cells undergoing the cell death pathway. As shown in Fig. 3, LDH levels at 24 hours post-infection showed small, albeit significant, increases after stimulation with wild-type bacteria at all inocula, compared to buffer alone. Similarly, wild-type *S. dysenteriae* 1 filtered supernatant and heat-killed bacteria also slightly affected cellular viability. Of note, the overall percentage of cell death observed after infection was never higher than 8% of positive control. Neither vaccine strains affected cell viability.

### *Shigella* infection affects epithelial paracellular permeability to macromolecules

**Figure 4:**
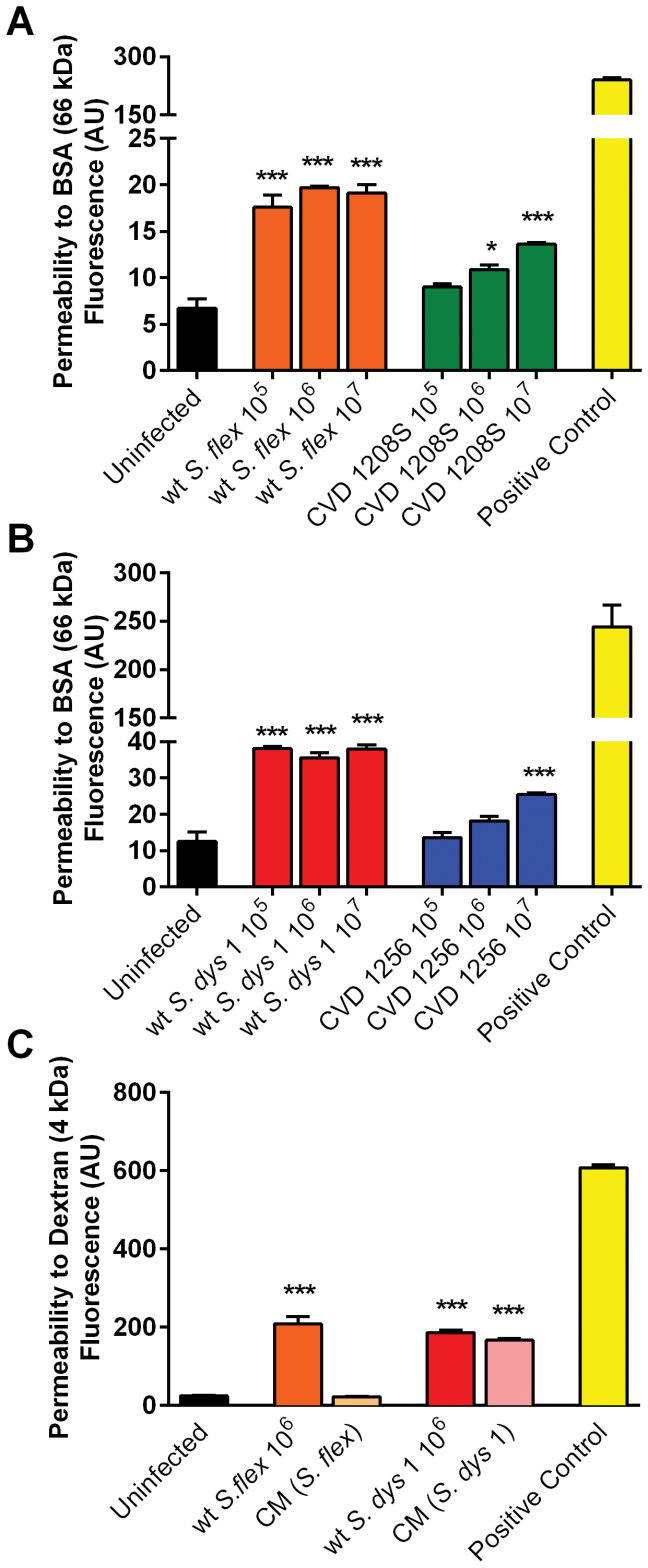
Wild type *Shigella* increase Caco2 monolayer paracellular permeability to macromolecules whilst vaccine candidates have no or markedly attenuated effect. **A**. FITC-BSA (66 kDa) net transport after infection with wild-type *S. flexneri* 2a or CVD 1208S applied at three different inocula. **B**. FITC-BSA (66 kDa) net transport after infection with wild-type *S. dysenteriae*-1 or CVD 1256 applied at three different inocula. C. FITC-Dextran (4 kDa) net transport after infection with wild-type filtered (Cond. Media: CM) or live *Shigella* applied at 10^6^ CFU. Calcium-free medium supplemented with EGTA to disrupt TJs served as positive control. Results are expressed as mean ± SEM of triplicate samples for each condition and are representative of 3 experiments with similar results. ***p<0.001, *p<0.05 compared to uninfected control (ANOVA).

We assessed alterations in barrier function following infection with wild-type *S. flexneri* 2a and *S. dysenteriae* 1 and their isogenic attenuated strains CVD 1208S and CVD 1256, respectively, by measuring the transepithelial flux of fluorescently labeled Dextran (FITC-Dextran, 4 kDa) or Bovine Serum Albumin (FITC-BSA, 66 kDa). As shown in Fig. 4, Caco2 cells exposed to wild-type *S. flexneri* 2a (Fig. 4A) or *S. dysenteriae* 1 (Fig. 4B) showed a significantly increased transport of FITC-BSA from the apical to the basolateral chamber compared to uninfected controls at all inocula evaluated. Consistent with the TEER data, the vaccine candidates CVD 1208S and CVD 1256 elicited a slightly increased permeability to BSA only when applied at the highest MOI. Permeability to FITC-dextran was increased as well when wild-type *Shigella* was applied to Caco2 monolayers and interestingly only conditioned media from wild-type *S. dysenteriae* 1 induced a response entirely comparable to live bacteria (Fig. 4C). EGTA was used as a maximum permeability control for its ability to completely open tight-junctions.

### Tight-junction disassembly in *Shigella* infected Caco2 monolayers

The data described above indicated that *S. flexneri* 2a and *S. dysenteriae* 1 affect epithelial barrier integrity by increasing Caco2 monolayer paracellular permeability, with some loss of cell viability. In a steady state status, the polarized epithelium maintains its relative impermeability to macromolecules through the correct functioning and integrity of intercellular tight junctions. We examined the effect of *Shigella* infection on Caco2 monolayer tight junction barrier by analyzing the tight junction-associated protein ZO-1 and the transmembrane proteins claudin-1 and occludin by immunofluorescence microscopy.

**Figure 5:**
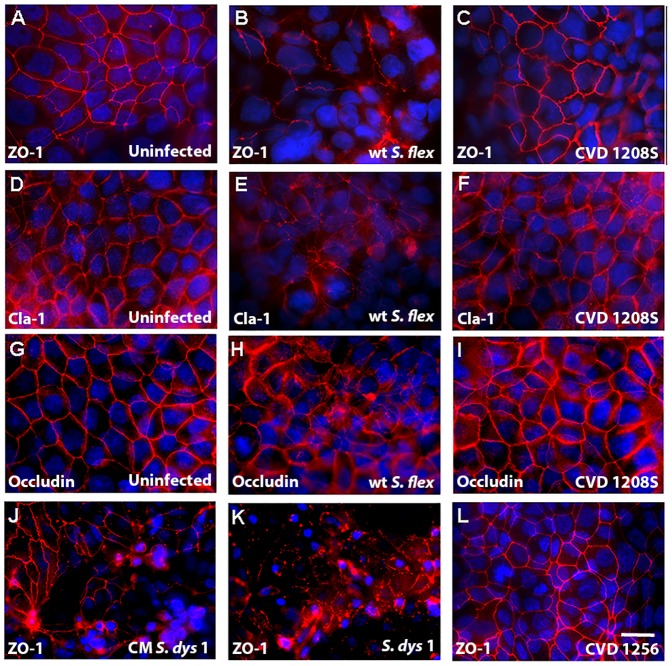
Tight-junction organization is disrupted following exposure to wild type *Shigella* but not to vaccine candidates. **A, D, G.** Uninfected monolayer immunostained for ZO-1, Claudin-1 or Occludin, respectively. **B, C.** Caco2 cells infected with wild-type *S. flexneri* 2a (B) or CVD 1208S (C) at 10^6^ CFU, stained for ZO-1 (1∶100). **E, F.** Caco-2 monolayers infected with wild-type *S. flexneri* 2a (E) or CVD 1208S (F) at 10^6^ CFU and stained for Claudin-1 (1∶1000). **H, I.** Monolayers infected with wild-type *S. flexneri* 2a (H) or CVD 1208S (I) at 10^6^ CFU and stained for Occludin (1∶100). **J, K, L.** Caco-2 cells treated with *S. dysenteriae* 1 filtered supernatant (J), live wild-type (K) or attenuated CVD 1256 (L) *S. dysenteriae* 1 strains applied at 10^5^ CFU and stained for ZO-1 (1∶100). Nuclei are stained with DAPI (blu). Bar 25 µm.

As shown in Fig. 5A, D, and G in uninfected monolayers ZO-1, claudin-1 and occludin are localized at the cell-cell boundary in a typical chicken wire-like pattern, indicating intact tight-junction complexes. In contrast, following infection with wild-type *S. flexneri* 2a at 10^6^ (Fig. 5B) we observed the complete loss of the ZO-1 chicken-wire patterning. ZO-1 appeared unevenly distributed throughout the monolayer without a proper organization, indicating the total loss of cell-cell attachment. In contrast, CVD 1208S applied at the same inoculum had no effect on ZO-1 organization (Fig. 5C). In monolayers infected with wild-type *S. flexneri* 2a at 10^6^, we observed a derangement of both claudin-1 and occludin distribution throughout the monolayer (Fig. 5E, H), compared to uninfected cells (Fig. 5 D, G) whereas no alteration in the localization of these tight-junction proteins has been observed in CVD 1208S infected cells (Fig. 5F, I). Similar results to those obtained for wild-type *S. flexneri* 2a and CVD 1208S were obtained for ZO-1 with wild-type *S. dysenteriae* 1 and the CVD 1256 isogenic attenuated strain applied at the same inoculum (data not shown). Figs. 5K and L show ZO-1 distribution following wild type S. *dysenteriae* 1 or CVD 1256 infection at the lowest bacterial load, respectively. Interestingly, *S. dysenteriae* 1 culture supernatant altered ZO-1 organization (Fig. 5J) although not as severely as viable bacteria (Fig 5K).

### *Shigella* infection induces phosphorylation of occludin and its disengagement from the tight junction complex

**Figure 6:**
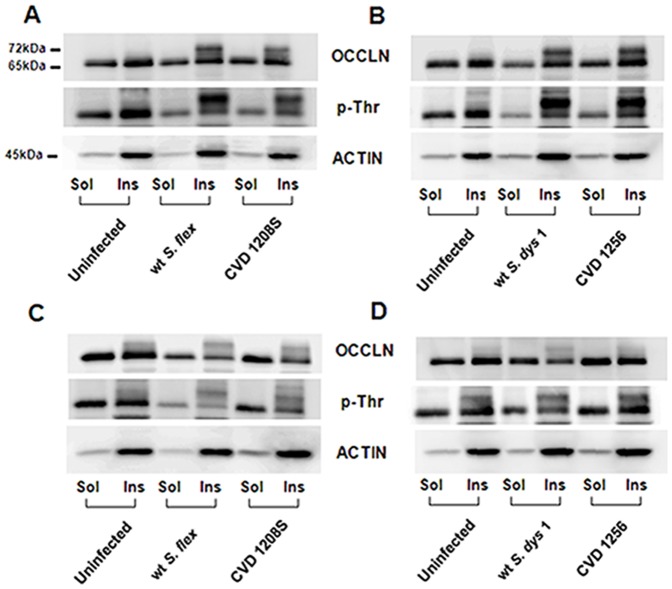
Occludin is hyperphosphorylated after infection with *Shigella* strains and disassembles from tight-junctions. **A**, **B**. Western blot of protein samples in the form of X-100 Triton soluble (Sol) and insoluble (Ins) fractions at 6 hours post-infection, blotted with anti-occludin (upper rows), anti-phosphothreonine (middle rows) and anti-actin (lower rows). **C**, **D**. Western blot of protein samples at 24 hours post-infection, blotted with anti-occludin (upper rows), anti-phosphothreonine (middle rows) and anti-actin (lower rows).

Occludin plays an important role in the regulation of tight-junction integrity [41]. It has been shown that overexpression of occludin results in TEER elevation [42], [43]. To understand the implication of occludin in the increased permeability caused by *Shigella* infection, we analyzed its solubility in the nonionic detergent Triton-X 100 in untreated controls and infected Caco2 monolayers (Fig. 6). In the untreated monolayer occludin was detected mostly in the insoluble fraction and was visible on western blots as a strong band of about 65 kDa [Low Molecular Weight (LMW) band; Fig. 6A, B upper panel]. Upon infection with wild-type *S. flexneri* 2a or *S. dysenteriae* 1 applied at 10^6^ we detected an additional band of about 72–79 kD in the insoluble membrane fraction at 6 hours post-infection. This high molecular weight (HMW) band was observed in both CVD vaccine strain-treated cell lysates, as well and represents a hyperphosphorylated form of occludin, specifically associated with the functional sealing components of tight-junctions [44]–[46]. It has been shown that occludin undergoes dephosphorylation on Ser/Thr residues during the disruption of tight junctions by various insults. Analysis of the Thr phosphorylation status shows that occludin is phosphorylated in the resting epithelium. Following bacterial infection occludin undergoes hyperphosphorylation on Thr (Fig. 6A, B middle panel) interestingly coincident with the raise in TEER observed at 6 hours. At 24 hours, we observed the loss of the hyperphosphorylated form of occludin together with a decrease of occludin in the membrane fraction, in the infected cells (Fig. 6C, D upper and middle panels). Both attenuated strains had a similar although milder effect on occludin.

### *Shigella* and attenuated strains induce a polarized secretion of IL-8

**Figure 7:**
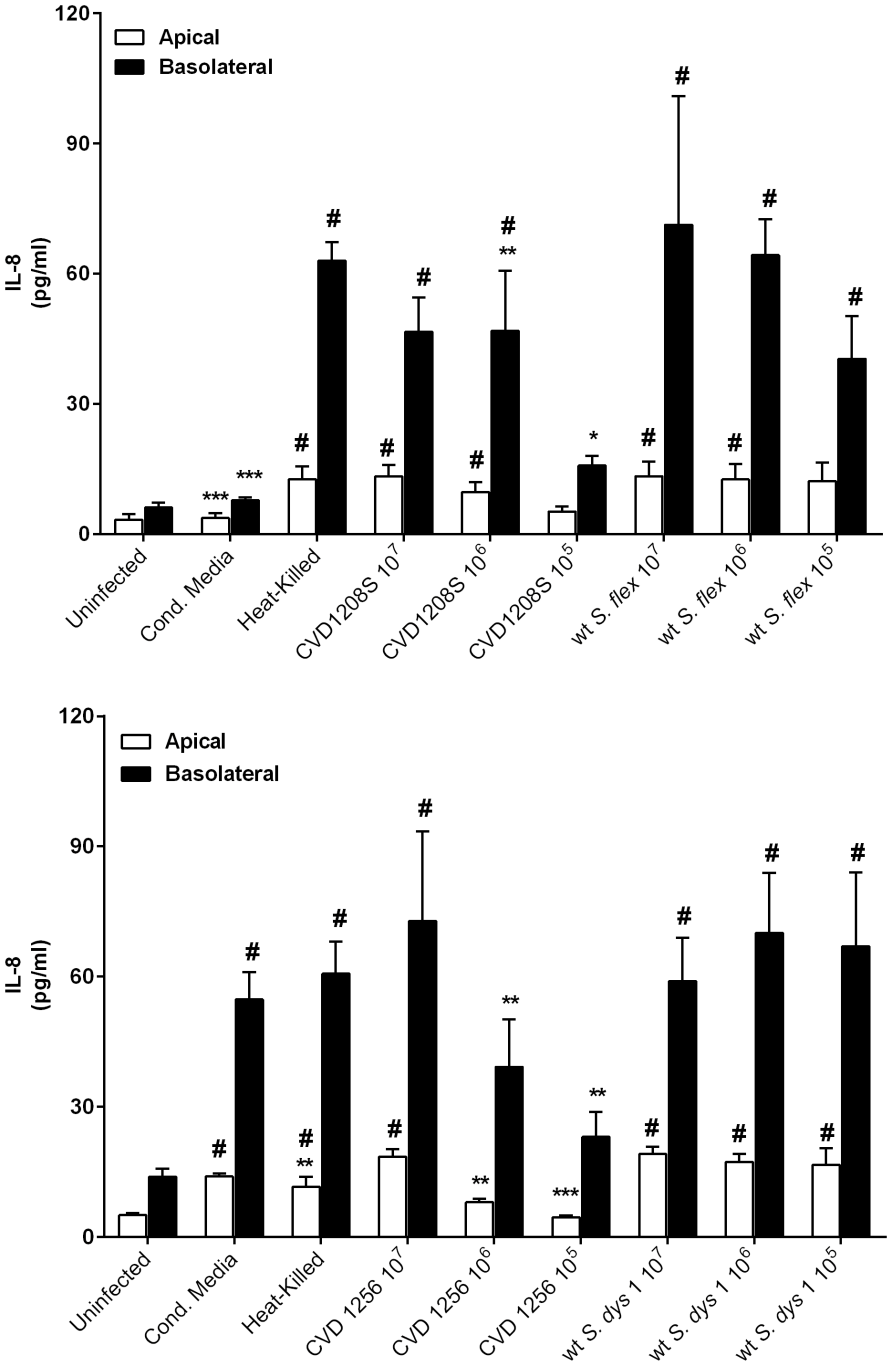
Wild type *Shigella* and attenuated strains induce Caco2 cells polarized secretion of IL-8. IL-8 secreted by Caco-2 cells infected with wild-type live, filtered and heat-killed *S. flexneri* 2a or vaccine candidate *S. flexneri* 2a CVD 1208S, applied at different inocula. **B**. IL-8 released by Caco2 cells infected with wild-type live, filtered and heat-killed *S. dysenteriae* 1 or *S. dysenteriae* 1 CVD 1256 applied at different inocula. Statistical comparisons are between infected and uninfected cells (**#**) or between the attenuated and the wild-type strains at the same inoculum (*). ***p<0.001, **p<0.01, * and #*p*<0.05 (ANOVA).

Cytokines analyzed in this study included IL-8, IL-6, IL-10, IL-12p70, IFNγ, IL1β and TNFα. With the exception of IL-8, all the other pro-inflammatory molecules were below detection level. IL-8 secretion was induced in Caco2 monolayers following infection with wild-type *S. flexneri* 2a and *S. dysenteriae* 1 and both vaccine candidate strains, at all inocula used in this study. Results from the analysis of media collected from the upper and lower chambers of transwells at 24 hours post infection are depicted in Fig. 7. As expected, we observed a polarized secretion of IL-8, predominantly in the basolateral side. Fig. 7A shows the levels of IL-8 measured in wild-type *S. flexneri* 2a and CVD 1208S infected monolayers. Overall, we observed a significantly higher level of IL-8 in cells infected with wild-type *Shigella* compared to the uninfected control, at all inocula evaluated. Likewise, IL-8 levels in wild type *Shigella* treated cells were significantly higher than CVD 1208S, except at the highest infection dose. Similarly, IL-8 basolateral levels in wild-type *S. dysenteriae* 1 treated cells were significantly higher than in uninfected controls at all inocula tested. CVD 1256 infected cells secreted a significant amount of IL-8 over uninfected control, only when applied at a MOI of 500 (10^7^). Basolateral IL-8 levels in CVD 1256 treated monolayers were significantly lower than wild-type *S. dysenteriae* 1 at bacterial infections of 10^6^ and 10^5^. Interestingly, both heat-killed wild-type *Shigella* strains induced high levels of IL-8 and, only for *S. dysenteriae* 1, the conditioned media, as well.

## Discussion

Shigellosis is typically characterized by two phases: an initial phase of watery diarrhea thought to arise in the jejunum, followed by a second phase characterized by stools containing blood, mucus and pus, as a result of bacterial invasion of the colonic epithelium. In milder cases, watery diarrhea may represent the only clinical presentation of the disease [47], [48]. Unlike other enterobacteria, ingestion of as low as 10 microorganisms are sufficient to cause shigellosis implying that *Shigella* must have evolved an array of tools in order to resist the low pH of the stomach and safely and efficiently travel through the small intestine ultimately reaching their final colonizing/replicating niche in the colon. Although the pathophysiology of *Shigella* infection has been widely studied, little is known about the early stage interaction of the pathogens with the epithelium of the small intestine. By using Caco2 cell monolayers, morphologically and functionally mimicking the small intestinal mucosal barrier [49]–[51], we aimed at getting insights into the biological response of the small intestine to *Shigella* infection.

Our data show that exposure to *S. flexneri* 2a and *S. dysenteriae* 1 cause a permanent impairment of the mucosal barrier integrity independent of the bacterial inocula and associated to a minimal, although significant loss of cell viability. We further analyzed the effect of *Shigella* on barrier integrity and we were able to provide molecular evidence showing that intercellular tight-junctions were disassembled (Fig. 5) and that changes in the phosphorylation status of occludin (Fig. 6) contribute to the impairment of the epithelial fence. Interestingly, while in *S*. Typhi infected monolayers early occludin hyperphosphorylation was paralleled by a drop in TEER (40), in Caco2 cells exposed to low inocula *Shigella* the change in the phosphorylation status of occludin appears to be consistently paralleled by a slight TEER increase (Fig. 1A, C), suggesting a transient enhancement of barrier function. While several studies have demonstrated that depending on the kinases and the specific occludin residues phosphorylated, distinct effects on tight junctions' assembly and function can be observed [52]–[57], more in depth studies are needed to clarify the molecular mechanisms behind this discrepancy. Our results indicate that infection of enterocytes with wild-type *Shigella* induces molecular changes in occludin, leading to its disengagement from the tight junction complex that, together with the associated disruption of claudin-1 and ZO-1 organization, contribute to increase paracellular permeability to macromolecules. Our analysis of the vaccine candidates CVD 1208S and CVD 1256, demonstrates that both have a general attenuated effect on barrier integrity and function, except when applied at the highest inocula. Interestingly, the application of heat-killed bacteria had little or no consequence on mucosal barrier function whereas *S. dysenteriae* 1 conditioned media had a severe effect on Caco2 monolayer integrity and permeability comparable to live bacteria, suggesting that while direct interaction with epithelial cells by viable *S. flexneri* is required to affect barrier function, *S. dysenteriae* 1 releases into the surrounding environment mediators whose activity alone strongly impairs the monolayer integrity.

A challenge in developing effective and safe vaccines is the ability to elicit protection with minimal or no inflammatory reaction. Epithelial cells have the capability to release and array of pro-inflammatory molecules, including IL-8. *Shigella* spp. were reported to induce secretion of IL-8 in colonic T84, HT29 or Caco2 cell monolayers [58]–[61]. We have now extended these findings by showing the polarized secretion of IL-8 upon apical exposure of Caco2 cell monolayers to wild-type *S. flexneri* 2a or *S. dysenteriae* 1 and their vaccine candidates. Heat-killed bacteria from both wild-type *Shigella* strains cause a release of IL-8 comparable to live organisms, suggesting that viable bacteria/epithelial cell interaction is not required for the induction of IL-8 secretion. Moreover, while exposure of Caco2 cells to *S. flexneri* 2a conditioned media does not elicit the secretion of IL-8, conditioned media from wild-type *S. dysenteriae* 1 is able to induce the secretion of high levels of IL-8, indicating that the presence of bacteria-secreted mediators alone is sufficient to trigger an immune response. Although release of IL-8 following infection with CVD 1208S and CVD 1256 vaccine candidates was lower than that observed with the wild-type parental strains, especially at lower bacterial infection inocula, they still induce a remarkable pro-inflammatory response compared to uninfected cells. At higher bacterial inocula, no difference in IL-8 levels was observed between wild-type and vaccine strains.

These data, together with the initial results obtained from clinical trials with CVD 1208S [39] and the encouraging immunological Sereny test results on guinea pigs upon infection with CVD 1256 [30], make these vaccine candidates' future application as effective, safe and capable of eliciting protective immunity vaccines, very promising.

Overall, we have not observed differences between the two wild-type *Shigella* strains in their ability to disrupt mucosal barrier integrity and permeability. The two vaccine candidates showed a much smaller number of bacteria in the monolayer compared to their isogenic parents, in line with their attenuated properties [30].

With the present studies we have provided for the first time mechanistic insights on the early interaction of *Shigella* species with the small intestine leading to increased permeability (including the mechanisms leading to tight junction disassembly), antigen trafficking, and cytokine release. Our data demonstrate that wild-type *Shigella* spp. affect mucosal barrier function and immune response and that these two events are unrelated, suggesting a differential (biphasic?) pathogenic effect along the intestinal tract. However, *S. dysenteriae* 1 secretes active mediator(s) (e.g., Shiga toxin) capable by themselves of inducing the same barrier alteration and immune response as viable bacteria. The lack of effect following exposure to *S. flexneri* 2a conditioned media suggests that the enterotoxins ShET1 and/or ShET2 alone do not play a role in barrier function alteration and inflammation. We have previously shown that ShET1 and 2 alter electrolyte and water transport in rabbit small intestine both *in vitro* and *in vivo* [5], [62], strongly suggesting their function as enterotoxins and a pivotal role in the onset of watery diarrhea in shigellosis. Recently, Faherty at al. [63] confirmed the enterotoxic activity of ShET 1 and 2 in an *ex-vivo* mouse model and identified a novel *S. flexneri* effector responsible for enterotoxic activity. All these studies have made use of highly concentrated supernatants from *Shigella* cultures. Since we have not tested either the amount or the activity of these toxins in our supernatants, we cannot exclude that they either have lost their activity during preparation or, more likely, are present in low concentrations, not sufficient to affect barrier function.

Nevertheless, data obtained following infection with the vaccine candidates, which are depleted of the major enterotoxins (ShET1/2 and stx), suggest that the increased paracellular permeability observed in Caco2 monolayers following exposure at the highest bacterial inoculum, might be attributed to a specific response of the host to infection rather than to bacterial toxins. The fact that attenuated *Shigella* strains induce a remarkable immune reaction, shows that the host immune response is not only independent of the functional impairment of the epithelial barrier but also of the bacteria main effector enterotoxic activities (ShET 1/2 and stx).

In summary, we speculate that wild-type *Shigella* infection causes a severe alteration of the small intestinal mucosa barrier function that might contribute to the induction of watery diarrhea as a mechanism for the host to eliminate harmful bacteria and for the pathogens to be efficiently transported to the large intestine where they invade the epithelium inducing a strong inflammatory response.
